# The Mentalized Affectivity Scale (MAS): Development and validation of the Italian version

**DOI:** 10.1371/journal.pone.0249272

**Published:** 2021-04-05

**Authors:** Teresa Rinaldi, Ilaria Castelli, Andrea Greco, David M. Greenberg, Elliot Jurist, Annalisa Valle, Antonella Marchetti

**Affiliations:** 1 Research Unit on Theory of Mind, Department of Psychology, Università Cattolica del Sacro Cuore, Milan, Italy; 2 Department of Human and Social Sciences, University of Bergamo, Bergamo, Italy; 3 Interdisciplinary Department of Social Sciences and Department of Music, Bar-Ilan University, Ramat Gan, Israel; 4 Clinical Psychology at the City College of New York, and The Graduate Center of the City University of New York, New York, NY, United States of America; Aalborg University, DENMARK

## Abstract

This study proposes a psychometric validation of the Italian version of the Mentalized Affectivity Scale (MAS) developed by Greenberg and colleagues in 2017. The mentalized affectivity construct integrates mentalization ability in the process of emotional regulation. An adult sample (N = 506) completed the 60-items MAS online version. In contrast to the three-factor structure of the original version, the Italian context confirmatory and exploratory factor analyses with splitted sample (CFA = 258; EFA = 248) revealed a five-factor structure. The hierarchically structured MAS factors are: Emotional Processing (being able to process emotion in situations); Expressing Emotions (talking and knowing emotions); Identifying Emotions (awareness of emotions); Control Processing (to control emotional reactions and expression), and Autobiographical Memory (related to childhood experiences). We also verified the convergent validity and reliability of the Italian version of the MAS by correlating the above five factors with measures of emotion regulation and reflective functioning. Moreover, we analyzed the relationships among the factors of the MAS, personality measures and well-being indexes, such as life satisfaction and self-efficacy: The new 35-item MAS scale showed robust correlations with all the tested constructs. Our results confirm that the MAS is a useful measure to assess mentalized affectivity, with the Italian version showing a more complex structure than the original English one, thus enriching the literature about mentalization.

## Introduction

Beginning from early childhood, people learn how to manage their emotions in everyday life in order to adapt appropriately to social situations [1]. This ability, known as emotion regulation, is defined as *“the extrinsic and intrinsic processes responsible for monitoring*, *evaluating*, *and modifying emotional reactions*, *especially their intensive and temporal features*, *to accomplish one’s goals*” [2]. It denotes psychological processes involved in the use of specific strategies, which aim at managing emotions. The clinical perspective has introduced a broader concept of affect regulation, a psychological construct with a biological base, consisting of a series of innate automatic mechanisms aimed at maintaining equilibrium with the environment [3], that contributes to the construction of attachment relationships to manage, alter, and change the affective state. Affect regulation relies upon the development of mentalization, because it involves the ability to reflect on one’s own and others’ emotional inner states and to be aware of such mental content during the modulation of the emotions [1, 4]. Mentalization is the ability to understand and interpret human behavior on the basis of mental states as intentions, emotions, desires, and beliefs. It has been described as *“the process by which a brain becomes a mind”* ([3], pg. 428). In its cognitive and affective components, mentalization is the source of adults’ social competences, supports interpersonal relationships and significantly contributes to manage social and relational situations [5]. Where mentalization is lacking, the individual acts without keeping others’ minds in mind, which produces social problems and leaves the individual feeling inadequate (as is the case, for example, with borderline personality disorder [6]. Mentalizing abilities have an impact upon affect regulation, thus facilitating the emergence of a more sophisticated kind of affect regulation, mentalized affectivity [4, 7].

Mentalized affectivity concerns a curiosity about emotions and refers to the adult ability to make sense of one’s own affective experience, activating reflection on it [7]. So, in mentalized affectivity, affect regulation is the capacity to become aware of one’s one affect by remaining within that affective state, and to attribute a meaning to that state by referring to past experiences, either real or imagined. Then, mentalized affectivity affirms an individual’s affective experience through representation of current and future experiences in light of the meaning attributed to past history. Just as mentalization plays an important role in the well-being and in personality construction [3], mentalized affectivity also contributes to the well-being of the individual. Jurist [7] affirms that mentalized affectivity helps to recognize meaningful events and situations, to deal with what happens and to facilitate communications, supporting the capacity to appreciate positive aspects of life and to promote the adaptation to different situations [8]. Moreover, autobiographical memory, the main aspect that differentiates mentalized affectivity from other constructs of emotion regulation, plays an important role in the construction of self-narratives, which are aimed to attribute new meanings regarding one’s existence. When this aim is achieved, the individual can construct a sense of self including all aspects of her/his experience; conversely, when it fails, there is a risk of various forms of psychopathology and personality disorders [9].

As described by Jurist [7] and elaborated by his research group (Greenberg et al. [1]), mentalized affectivity consists of three components: “Identifying emotions”, “Processing emotions”, and “Expressing emotions”. “Identifying emotions” does not only mean being able to recognize and to name emotions, but also becoming aware of their meaning in the situations in which they occur or, subsequently when rethinking about past experiences. “Processing (or modulating) emotions” means knowing how to manage emotions, for example modifying their intensity, or refining them in the light of new experiences. Finally, “Expressing emotions” refers to two levels, one related to inward expression, and one related to communication to others. The first level requires the concept of reflective functioning and presumes that the individual is able to experience her/his own emotions fully, without necessarily revealing them to others. The second level refers to the capacity to communicate one’s own internal states, considering others’ internal world and receptivity. In the latter case, “Expressing emotions” means being able to help other people to be able to understand and be responsive to what we feel, both implicitly and explicitly, by verbalizing them, describing or simply acknowledging their disclosure.

Recently, emotion regulation and mentalization have been the subject of interest in cross-cultural studies and reflections. Regarding emotion regulation, Ford and Mauss [10] consider the classic distinction between cultures promoting interdependence (Eastern cultures) and cultures promoting independence (Western cultures) and highlight that cultures play an important role in determining the motivation of individuals’ emotion regulation and in the use of specific strategies in different regulatory contexts. In interdependent cultures, individuals prefer to regulate their emotions as a way to maintain collective harmony, using strategies like expressive suppression. On the contrary, in independent cultures, expressive suppression is not considered an adaptive strategy, because individuals are less motivated to be concerned with collective harmony. Although studies about cultural differences in mentalization are still limited, in a recent review Aival-Naveh and colleagues [11] reflect upon the difference between individualistic *vs*. collectivistic cultures: in the former, mentalization is mainly self-oriented, whereas in the latter, mentalizing abilities develop firstly with the aim to understand others, then they are applied to understand themselves. Commenting on this review, Fonagy and Campbell [12] suggest that this is due to the different way in which attachment bonds are constructed. In individualistic cultures, parenting practices require that the newborn interact mainly with the caregiver, whereas in collectivistic cultures, the caregiver welcomes the infant to interact with all the members of the community; in the former, the baby is a primary focus of the caregiver’s mind and, consequently, of her/his own, in the latter, the focus is on other people’s minds and the ability to interact with them. In spite of the limited number of available studies, Jurist and Sosa [13] argue for the importance of identifying cross-cultural differences in mentalization and particularly in mentalized affectivity, a complex construct that, connecting emotion regulation, mentalization and autobiographical memory, is likely to be strongly influenced by the culture to which it belongs.

### How to measure mentalized affectivity?

In order to evaluate the three components of mentalized affectivity, Identifying emotions, Processing emotions, and Expressing emotions, Greenberg and colleagues created the *Mentalized Affectivity Scale*—MAS ([1], Italian translation in [14]), a 7-points Likert scale in which respondents indicate their degree of agreement to 60 statements. A principal-components analysis (PCA) with varimax rotation showed that the Kaiser-Meyer-Olkin Measure of Sampling Adequacy was .95, and that the 60-items scale explained the 43% of the variance [1]. Moreover, the hierarchical analysis revealed a three-factors scale structure: Identifying, Processing, and Expressing. Examples of the Identifying-factor items are: “Understanding my emotional experience is an ongoing process” or “I am curious about identifying my emotions”. For the Processing-factor, some items are “When I am filled with a negative emotion, I know how to handle it” or “I am good at controlling my emotions”. Finally, for the Expressing-factor, some examples are “People tell me I am good at expressing my emotions” or “I often keep my emotions inside”.

As it can be seen from the examples above, answering the items of the MAS requires a mentalizing about one’s own positive and negative emotions. This process focuses both on one’s own personal experience in emotion management (e.g. “I am good at distinguishing between different emotions that I feel”), and on the tendency to take the point of view of other people in relation to oneself (e.g. “I am open to other people’s view of me because it helps me to better understand myself”). Due to the complexity of the mentalization process concerning one’s own emotions, Greenberg and colleagues [1] investigate the characteristics of the MAS also in a sample with psychological disorders, showing that this scale individuates significant differences between a typical and atypical population. Comparing a typical sample with a sample characterized by 18 clinical diagnoses, the authors demonstrate that in people with psychological disorders, the score in the Identifying factor is higher compared to the typical population; on the contrary, the score in the Processing factor is lower in people with psychological disorders than in the typical population. Although the authors do not identify a cut-off score, the MAS seems to discriminate some relevant components of mentalized affectivity in a clinical population, offering significant considerations with respect to the type of treatment useful to these subjects in dealing with emotions. Research is now being conducted using a clinical population.

The involvement of mentalization in emotion regulation made the MAS scale an innovative tool in the international panorama, with translations into 11 different languages [13]. In fact, as already highlighted by Greenberg and colleagues [1], several tasks assessing other constructs close to mentalized affectivity have been created over the past years, but they are able to capture only certain aspects of the larger construct of mentalized affectivity. For example, the *Emotion Regulation Questionnaire* (ERQ; [15] in the Italian version of [16] is a 10 items tool on a 7-point-Likert scale detecting the use of two different emotion regulation strategies: cognitive reappraisal, rethinking a situation in order to modify its emotional meaning, and emotional impact and expressive suppression, referring to modifying or reducing emotional behavior. In this case, there are some similarities between the cognitive reappraisal tested by the ERQ and the Processing factor of the MAS, as well as between the expressive suppression factor of the ERQ and the negative pole of the Expressing factor of the MAS, but the component of the Identification factor is lacking in the ERQ while it is present in the MAS. Well known measures developed so far to assess mentalization are the Reflective Functioning Scale [17], based on the Adult Attachment Interview, and the Reflective Functioning Questionnaire (RFQ), the first self-report measure developed to specifically assess one’s own mentalization ability [18].

These measures test mentalization in terms of reflective functioning, whereas the MAS emphasizes mentalized affectivity. In fact, although reflective functioning and mentalized affectivity are overlapping constructs, as they both imply the ability to reflect on oneself, the first one seems to regard mainly the reinterpretation of the past during critical life situations [19], whereas the second one is focused on the capacity to utilize live current emotional experience. This difference is also evident analyzing the structure of the RFQ, which has two subscales, Certainty and Uncertainty in mentalization. High scores on the “Certainty” subscale are related to hypermentalizing in reflective functioning, i.e. an “over-mentalizing” attitude where the attributed mental states do not correspond to reality. High scores on the “Uncertainty” subscale lead to hypomentalizing, which indicates a poor understanding of one’s own and others’ mental states [20]. So, the RFQ seems to be particularly sensitive to assess the distortions of mentalization [21], whereas the MAS aims at capturing mentalization along the continuum of typical and atypical functioning. Therefore, we have concluded that the development of an Italian version of the MAS is desirable, in order to have a useful tool for research and intervention on mentalization in the Italian context along with the other above-mentioned measures already developed in the past years.

### Aims

In the light of the increasing interest in mentalized affectivity and in its evaluation, we aimed to test the psychometric validity and the reliability of the Italian version of MAS in a cohort of Italian adults. Specifically, we aimed to:

1. test the factorial validity (with confirmatory factor analysis—CFA) and the hierarchical structure of the model proposed by Greenberg et al. [1]. We hypothesized that the Italian version of the MAS would reduce into three distinct factors based on the mentalized affectivity theory, as in the original version of the scale;
2. test the assessment’s reliability and concurrent and convergent validity by examining associations with the MAS and its socio-affective correlates: the emotion regulation, tested with the Emotion Regulation Questionnaire, and the reflective functioning, tested with the Reflective Functioning Questionnaire. In fact, according to the theoretical model, emotion regulation and reflective functioning are two constructs really close to the mentalized affectivity. Notably, we chose the ERQ in line with the theoretical proposal of Greenberg and colleagues [1], who state that the MAS assesses the emotion regulation, despite the existence of relevant differences between the two tasks in terms of awareness and mentalization of emotions. Regarding the relation between the MAS and the RFQ, given that both constructs involve mentalization skills (according to the mentalized affectivity model), we hypothesize the existence of associations between these two competences;
3. examine the psychological correlates of mentalized affectivity including personality measures and well-being (such as life satisfaction and self-efficacy). In line with the results obtained by Greenberg and colleagues (2017), we hypothesized that high levels of mentalized affectivity may correlate with some personality traits, such as openness to experience and extraversion (both related to the emotional experience), and with high levels of well-being.

## Methods

### Participants

The total number of participants was 779. The final sample comprised only those who completed 80% of the survey. There were 506 participants (223 (44.1%) were male) aged between 18 and 69 years (*M* = 31.8 years (*SD* = 13.4 years). The number of participants is lower than the original paper (*N* = 2,840; Greenberg et al., [1]), with similar characteristics with regards to gender (male = 901, 42%) and age (mean age = 31.58; SD = 11.90; range 18–65 years). The two samples differ in the level of education: in the Italian sample, most of the participants report a high school educational level (*N* = 336, 66.4%), in the original sample there are more distribution into the educational levels, with the most elevated percentage of people completed a graduate school (N = 806, 25.4%). Italian participants were mostly employed (*N* = 323, 63.8%), single (*N* = 362, 71.5%) and living with relatives (*N* = 252, 49.8%). Other sample characteristics are presented in Table 1. The only inclusion criteria to take part to data collection was to be on a legal age, i.e. over 18 years.

**Table 1:** Sample characteristics.

Sociodemographic characteristics			
Age, mean ± SD	31.8 ± 13.4	Employment status	N (%)
Gender	N (%)	Employed	323 (63.8)
Male	223 (44.1)	Unemployed	81 (16.0)
Female	283 (55.9)	Homemaker	12 (2.4)
Educational level	N (%)	Retired	15 (3.0)
No title	2 (0.4)	Retired with some work activities	1 (0.2)
Primary school	1 (0.2)	Student	74 (14.6)
Middle school	37 (7.3)	Residence type	N (%)
High school	336 (66.4)	Only with spouse or partner	60 (11.9)
Graduate school	104 (20.6)	With spouse or partner and children	96 (19.0)
Postgraduate school	26 (5.1)	By themselves	51 (10.1)
Marital Status	N (%)	Only with children	8 (1.6)
Single	362 (71.5.)	Only with other family members	252 (49.8)
Married	128 (25.3)	In a protected structure	1 (0.2)
Divorced/Separated	14 (2.8)	Other	38 (7.5)
Widowed	2 (0.4)		

### Procedures

Data were collected through an online survey hosted on the Qualtrics platform from February 2018 to January 2019. Once the study protocol was implemented and completed, a link to the survey was presented to university courses in the Psychology at the Department of Human and Social Sciences of the University of Bergamo, and of the Faculty of Education of the Catholic University of the Sacred Heart of Milan. The same link was sent to personal contacts and to other contacts of the participants through a snowball sampling method. In addition to providing a link to the survey, participants were presented with all the necessary information, including the purpose of the study, the instructions, the duration of the survey, which was estimated in about 30 minutes. In the first page of the survey, participants were informed about personal data processing, and only those who gave their informed consent were included in the data collection. Furthermore, all participants were treated in accordance with the ethical guidelines for research provided by the Declaration of Helsinki [22], American Psychological Association [23] and by Italian Psychological Association [24]. The study was approved by the local ethical committee of the Department of Psychology of the Catholic University of the Sacred Heart of Milan, according to APA ethical standards. Participants provided some socio-demographic information first, then they completed the Mentalized Affectivity Scale in the Italian translation provided in Jurist [11]. In order to test the validity of the scale, other questionnaires concerning personality, emotional regulation, perception of satisfaction with life, self-efficacy and reflective function were included.

### Measures

#### Sociodemographic information

All participants were asked to provide sociodemographic information such as gender, year of birth, education level, marital status, employment status, and residence type.

#### Emotion regulation

The *Emotion Regulation Questionnaire*. ERQ [25] in the Italian translation by Balzarotti and colleagues [16], is a self-report scale that evaluates the emotional regulation of participants. It is a 7-point Likert scale from 1 (I strongly disagree) to 7 (I strongly agree) consisting of 10 items representing the emotional regulation strategies of cognitive reappraisal (6 items) and expressive suppression (4 items) [16]. Scoring is obtained by creating an overall score from the two scores obtained in the subscales. The minimum and the maximum scores range from 10 to 70. Cronbach’s α for the cognitive reappraisal subscale is 0.847, while Cronbach’s α for the suppression subscale is 0.747.

#### Reflective functioning

The *Reflective Functioning Scale*. RFQ; [26] in the Italian version retrieved from the Psychoanalytic Unit of University College of London by Fonagy https://www.ucl.ac.uk/psychoanalysis/research/reflective-functioning-questionnaire-rfqme).

The short version of the scale was used: an 8-items self -report scale assessing reflective functioning from 1 (strongly disagree) to 7 (strongly agree). This scale has two scales: Certainty and Uncertainty in mentalization, evaluated on a 7-point Likert scale. Scoring is obtained summing up the items belonging to the two scales, 6 for Certainty (range 0–18) and 6 for Uncertainty (range 0–18). Cronbach’s α for the Certainty subscale is 0.689, while Cronbach’s α for the Uncertainty subscale is 0.656.

#### Personality

Personality has been assessed through the *Ten Item Personality Inventory*. TIPI [27], in the Italian version of Chiorri and colleagues (I-TIPI;25). The Italian version of the scale was freely downloaded from Samuel Gosling’s website (http://homepage.psy.utexas.edu/homepage/faculty/gosling/scales_we.htm). The I-TIPI is a self-report scale that investigates five dimensions of personality. The scale is developed using descriptors from Big Five instruments. The five personality dimensions are [28]: Extraversion (E), being able of preserving the species reproduction thanks to the ability to adapt to the social contexts; Agreeableness (A), having an optimistic view of human nature and get along well with people; Conscientiousness (C), being able to arrange personal things, be methodical and considered by others reliable; Neuroticism (N), related to anxiety and depression, defined as emotional instability, and Openness to Experience (O), be willing to experience with new things and have many and varied interests [29]. Each dimension consists of two items, in a total of 10 items with a 7-point Likert scale from 1 (strongly disagree) to 7 (strongly agree). The scoring is calculated by summing the two items for each factor. The minimum and the maximum scores range from 2 to 14. Cronbach’s α for each factor is: Extraversion α 0.661; Agreeableness α 0.199; Conscientiousness α. 0.456; Neuroticism α 0.496 and Openness to Experiences α 0.457.

#### Life satisfaction

*Satisfaction with Life Scale*. SWSL [30] in the Italian version of Di Fabio and colleagues [31] is a self-report scale that assesses respondents’ perception of satisfaction with their lives. It is 5-items scale designed to measure global cognitive judgments of one’s life satisfaction. Participants indicate how much they agree or disagree with each of the 5 items using a 7-point scale that ranges from 1 (I strongly disagree) to 7 (I strongly agree). The scoring is obtained by summing the scores of each of the 5 items and it ranges from a minimum of extreme dissatisfaction [5] to a maximum of extreme satisfaction [35] Cronbach’s α 0.855.

#### Self-efficacy

*General Self-Efficacy*. GSE [32] in the Italian version of Sibilia, Schwarzer, Jerusalem [33] evaluated through a self-report scale the perception that subjects have of their sense of self-efficacy referring to personal agency. It has 10 items on a 4-point Likert Scale from 1 (not all true) to 4 (exactly true). Scoring is evaluating summing up all the answers, from a minimum score of 10 to a maximum score of 40. Cronbach’s α 0.868.

## Statistical analysis

Data analyses were performed using Jamovi statistical software [The Jamovi project (2020). Jamovi (Version 1.2) (Computer Software). Retrieved from https://www.jamovi.org]. For the sample characteristics, mean values and standard deviations (SDs) for continuous variables were calculated; for categorical/nominal variables, frequencies and percentages were computed. Skewness and kurtosis of the MAS items were first checked to assess normal distribution; West, Finch, & Curran [34] recommend concern if skewness > 2 and kurtosis > 7.

The factorial validity of the MAS, considering the model proposed by Greenberg et al. [1], was assessed with confirmatory factor analysis (CFA). Hu and Bentler’s guidelines [35] for various fit indices were used to determine whether the expected model fits the data. The chi-square test statistic was employed, but considering its sensitivity to sample size, other fit indices were evaluated: (a) the comparative fit index (CFI ≥0.90 indicates a good fit); (b) the root mean square error of approximation (RMSEA ≤0.08 indicates an acceptable fit); and (c) the standardized root mean square residual (SRMR ≤0.08 indicates an adequate fit).

As is often the case, scales translated in different languages and analyzed in different cultural contexts, may not have the same latent factor structure of the original version: in this case, it is appropriate to examine the latent structure of the assessment through an exploratory factor analysis (EFA), followed by a new confirmative factor analysis (CFA). Since this is the case of this study, we examined the latent structure of the MAS through an exploratory factor analysis (EFA), followed by a new confirmative factor analysis (CFA). The total sample was later randomly divided into two halves. The first sample was used to perform an EFA (SAMPLE A, n = 258), and the second was used to perform a CFA in order to validate the EFA structure (SAMPLE B, n = 248).

On Sample A, the Kaiser Meyer Olkin (KMO) and the Bartlett’s test of sphericity were run in order to be sure that the correlation matrix could be subjected to analyses (KMO should be > 0.5; Bartlett’s test of sphericity should be significant). The Cattell scree test (judging the elbow of a scree plot) and the Kaiser-Guttman criteria (eigenvalue greater than one) were used to identify the number of factors to be extracted using EFA. EFA with the Oblimin oblique rotation was used to analyze the items on the MAS. Oblique rotation was used because the factors extracted from the MAS are likely to correlate with each other. In the first step, all 60 items were included. Subsequent factor analyses were conducted in a stepwise fashion, in order to eliminate items until a stable factor solution emerged. Loadings in the .40 range or above are generally considered the cut-off on substantial loadings [36, 37]; for this reason, items that had a factor loading <|.40| were excluded, and, after the first step, items that loaded at >|.40| on more than one factor were excluded. Moreover, in order to obtain a more refined and clear-cut solution, we selected those items that showed a loading higher than |.40| on the intended factor, but also a ratio higher than 2 among the primary loading and the highest secondary loading (i.e., the primary loading was two times the highest secondary loading).

On Sample B, CFA was conducted. Maximum Likelihood (ML) was used as an estimation method. Hu and Bentler’s guidelines for various fit [35] indices were used to determine whether the expected model fit the data.

Cronbach’s alpha coefficients were performed on the total sample to examine internal consistency. Cronbach’s Alpha below .60 are unacceptable [38].

To examine the hierarchical structure of the scale, the one-component through five component solutions was explored using the procedure proposed by Goldberg [39]. First, a single component was specified in a PCA and then, in four subsequent PCAs, we specified two, three, four, and five orthogonally rotated components. The component scores were saved for each solution. Next, correlations between component scores at adjacent levels were computed.

The concurrent validity of the MAS scale was evaluated by correlating the MAS factors with age, education, personality, emotion regulation, life satisfaction, self-efficacy and reflective functioning with the Pearson’s r correlation coefficient. Following Cohen’s guidelines [40] we interpreted correlations as measures of the effect size. Correlations were considered weak (|0.10| < r < |0.29|), moderate (|0.30| <r <|0.49|) or strong (|0.50| < r < |1|). Furthermore, t-tests were used to test the difference among profiles of the MAS factors due to gender. Missing values were treated via listwise deletion.

## Results

### Descriptive analysis of MAS items

The descriptive analysis of the MAS items is presented in Table 2. The average scores of the responses to the 60 items from all the participants ranged from to 60 to 420 and were split into three factors scores (Expressing from 14 to 98; Identifying from 24 to 168 and Processing from 22 to 154) (SD MIN = 1.15–SD MAX = 1.38). Moreover, in line with recommendations by Bulmer [41], the results showed that all items had a normal distribution (skewness MIN = −1.56 skewness MAX = 0.83; kurtosis MIN = −1.26–kurtosis MAX = 2.68).

**Table 2:** Mean, standard deviation, skewness and kurtosis of the 60-item MAS version.

	MEAN	STANDARD DEVIATION	SKEWNESS	KURTOSIS
**1. I often think about how the emotions that I feel stem from earlier life experiences (e.g., family dynamics during childhood).**	5.27	1.40	-1.10	0.70
**2. I can express my emotions clearly to others**	4.61	1.71	-0.48	-1.01
**3. I am good at understanding other people’s complex emotions.**	5.39	1.28	-1.11	1.01
**4. I use tools i have learned to help when I am in difficult emotional situations**	5.11	1.40	-0.92	0.42
**5. I can see how prior relationships influence my current emotions.**	5.60	1.22	-1.26	1.68
**6. I can still think rationally even if my emotions are complex.**	4.97	1.58	-0.69	-0.42
**7. I am able to wait to act on my emotions.**	4.62	1.69	-0.40	-0.90
**8. I put effort into managing my emotions.**	5.29	1.42	-1.19	1.07
**9. It is hard for me to talk about my complex emotions.**	4.88	1.82	-0.63	-0.77
**10. When I am filled with a negative emotion, I know how to handle it**	4.17	1.61	-0.22	-1.06
**11. I often know the reasons why I feel the emotions I do.**	5.17	1.44	-0.92	0.14
**12. Understanding my emotional experience is an ongoing process.**	5.72	1.29	-1.14	1.11
**13. I am often confused about the emotions that I feel.**	3.59	1.75	0.27	-1.08
**14. I am able to adjust my emotions to be more precise.**	3.71	1.54	-0.05	-0.75
**15. It is hard for me to manage my emotions.**	3.75	1.68	0.16	-1.05
**16. Knowing about my childhood experiences helps to put my present emotions within a larger context.**	4.76	1.56	-0.55	-0.48
**17. It is easy for me to notice when I am feeling different emotions at the same time.**	4.71	1.40	-0.57	-0.23
**18. I often think about my past experiences to help me understand Emotions that I feel in the present.**	5.11	1.44	-0.92	0.30
**19. I am able to keep my emotions to myself if the timing to express them isn’t right.**	5.26	1.70	-0.93	-0.10
**20. I often keep my emotions inside.**	4.96	1.79	-0.65	-0.77
**21. I can easily label “basic emotions” (fear, anger, sadness, joy, and surprise) that I feel.**	5.68	1.34	-1.30	1.52
**22. I am good at increasing emotions that I want to feel more.**	3.93	1.61	-0.01	-0.83
**23. I am good at controlling my emotions.**	4.56	1.62	-0.46	-0.85
**24. When I express my emotions to others, it is usually jumbled.**	3.89	1.77	0.04	-1.20
**25. When I am filled with a positive emotion, I know how to keep the feeling going.**	4.35	1.45	-0.20	-0.50
**26. I am good at controlling emotions that I do not want to feel.**	3.29	1.69	0.55	-0.73
**27. I am quick to act on my emotions.**	4.23	1.63	-0.15	-0.90
**28. It helps me to know the reasons behind why I feel the way that I do.**	5.57	1.26	-1.23	2.02
**29. I am aware of recurrent patterns to my emotions.**	5.38	1.32	-1.14	1.25
**30. People tell me I am good at expressing my emotions.**	4.07	1.61	-0.11	-0.62
**31. If I feel something, I prefer not to discuss it with others.**	4.00	1.75	-0.03	-1.11
**32. It takes me a while to know how I am really feeling.**	3.86	1.71	-0.01	-1.15
**33. I try to understand the complexity of my emotions.**	5.12	1.34	-0.73	0.26
**34. It is important for me to acknowledge my own true feelings.**	5.97	1.20	-1.56	2.68
**35. I often figure out where my emotions stem from.**	5.09	1.36	-0.83	0.21
**36. If I feel something, I would rather not convey it to others.**	4.41	1.67	-0.24	-0.88
**37. I often look back at my life history to help inform my current emotional state and situation.**	5.14	1.45	-0.74	-0.034
**38. I am open to what others say about me to help me know what I am feeling.**	5.08	1.43	-0.79	0.037
**39. People get confused when I try to express my emotions.**	3.43	1.53	0.31	-0.61
**40. Sometimes it is good to keep my emotions to myself.**	5.51	1.38	-1.09	0.94
**41. I am good at distinguishing between different emotions that I feel.**	5.14	1.32	-0.86	0.28
**42. I am curious about identifying my emotions.**	5.32	1.36	-0.70	-0.02
**43. If a feeling makes me feel uncomfortable, I can easily get rid of it.**	3.38	1.60	0.42	-0.80
**44. I often know what I feel but choose not to reveal it outwardly.**	4.88	1.57	-0.55	-0.51
**45. If I feel something, it often comes pouring out of me.**	3.82	1.75	0.11	-1.12
**46. I try to put effort into identifying my emotions.**	5.06	1.42	-0.75	0.08
**47. I can pinpoint childhood experiences that influence the way that I often think and feel.**	5.04	1.55	-0.71	-0.20
**48. If I feel something, I will convey it to others.**	4.22	1.57	-0.31	-0.73
**49. Thinking about other people’s emotional experiences helps me to think about my own.**	4.87	1.56	-0.85	0.002
**50. I can see how prior relationships influence the relationships that I have now.**	5.37	1.33	-1.00	0.77
**51. It is helpful to think about how my emotions stem from family dynamics.**	5.34	1.42	-1.14	1.06
**52. I am open to other people’s view of me because it helps me to better understand myself.**	5.17	1.39	-0.87	0.34
**53. I rarely think about the reasons behind why I am feeling a certain way.**	2.96	1.65	0.80	-0.28
**54. It’s important to understand the major life events that have had an impact on my behavior.**	5.75	1.15	-1.32	2.28
**55. I am not aware of the emotions I’m feeling when in conversation.**	2.67	1.46	0.83	-0.07
**56. I am more comfortable “talking around” emotions I am feeling, rather than talking about them directly**	3.89	1.82	0.05	-1.26
**57. I can quickly identify my emotions without having to think too much about it.**	4.58	1.56	-0.46	-0.74
**58. I am able to understand my emotions within the context of my surroundings.**	5.05	1.24	-0.70	0.19
**59. I can tell if I am feeling a combination of emotions at the same time.**	4.90	1.31	-0.74	0.23
**60. I am interested in learning about why I feel certain emotions more frequently than others.**	5.58	1.27	-1.16	1.34

### Confirmative factor analysis

A confirmative analysis with varimax rotation was run using Greenberg and colleagues’ criteria [1, 42]. The CFA fits statistics of the three factors model exhibited a poor fit (χ2(1710) 5337,50, P≤0.001; CFI 0.60; RMSEA 0.07; SRMR 0.12).

### Factor structure of the Mentalized Affectivity Scale. Exploratory factor analysis

Data from Sample A and 60 items were used in these analyses. The Bartlett’s sphericity test (χ2 = 7605, p < .001) and the KMO = 0.84 have ensured that the correlation matrix could be subjected to factor analysis. The Cattell scree test and the Kaiser-Guttman criteria indicated that a five-factor solution was the most appropriate. EFA was then conducted, with five factors extracted. The initial pool of 60 general items, after subsequent factor analyses conducted in a stepwise fashion, was reduced to 35. The following twelve items were excluded, because their loadings were lower than .40: “I am good at understanding other people’s complex emotions.”; “I use tools I have learned to help when I am in difficult emotional situations.”; “I can see how prior relationships influence my current emotions.”; “I put effort into managing my emotions.”; “It is easy for me to notice when I am feeling different emotions at the same time.”; “I am good at increasing emotions that I want to feel more.”; “When I am filled with a positive emotion, I know how to keep the feeling going.”; “I am quick to act on my emotions.”; “I am aware of recurrent patterns to my emotions.”; “I am open to what others say about me to help me know what I am feeling.”; “Thinking about other people’s emotional experiences helps me to think about my own.”; “I am open to other people’s view of me because it helps me to better understand myself”.

The following thirteen items were excluded because they showed a ratio higher than 2 among the primary loading and the highest secondary loading: “I can express my emotions clearly to others.”; “It is hard for me to manage my emotions.”; “I often think about my past experiences to help me understand emotions that I feel in the present.”; “I am able to keep my emotions to myself if the timing to express them isn’t right.”; “People tell me I am good at expressing my emotions.”; “I often look back at my life history to help inform my current emotional state and situation.”; “People get confused when I try to express my emotions.”; “I can see how prior relationships influence the relationships that I have now.”; “It is helpful to think about how my emotions stem from family dynamics.”; “I rarely think about the reasons behind why I am feeling a certain way.”; “It’s important to understand the major life events that have had an impact on my behavior.”; “I am more comfortable “talking around” emotions I am feeling, rather than talking about them directly.”; “When I express my emotions to others, it is usually jumbled”.

The pattern of factor loadings from the five-factors exploratory measurement model for the MAS scale with 35 items is given in Table 3. The first extracted factor explains 12.85% of the variance after rotation. It showed loadings from ten items evaluating a self-assessment of one’s ability to be aware of one’s own emotions. This factor can be named “Identifying Emotion”. The second extracted factor explains 10.64% of the variance after rotation. It showed strong loadings from eight items assessing the way people try to express and communicate their emotions with others, i.e. externalizing them. This factor can be labelled “Expressing Emotions”. The third extracted factor explains 9.53% of the variance after rotation. It showed loadings from seven items assessing people’s ability to identify and label their emotions. This factor can be called “Curiosity about Emotions”. The fourth extracted factor explains 7.80% of the variance after rotation. It showed strong loadings from seven items assessing people’s ability to control their emotions using cognition. This factor can be named “Processing Emotions”. The fifth extracted factor explains 4.89% of the variance after rotation. It showed loadings from three items assessing people memories about personal childhood emotion experiences. This factor can be labelled “Autobiographical Memory”. The total variance explained by the five factors extracted was 45.7%. As shown in Table 2, no item displays a loading lower than .40. The extent of cross-loading between factors was moderate; the size of this secondary loading was usually small, below .30.

**Table 3:** Component loadings for 35-item MAS.

	Factors					
	1	2	3	4	5	Stnd Est.
**41. I am good at distinguishing between different emotions that I feel.**	0.783					0.772
**57. I can quickly identify my emotions without having to think too much about it.**	0.759					0.725
**35. I often figure out where my emotions stem from.**	0.709					0.691
**58. I am able to understand my emotions within the context of my surroundings.**	0.697	-0.274				0.739
**32. It takes me a while to know how I am really feeling.**	0.647					-0.584
**11. I often know the reasons why I feel the emotions I do.**	0.589					0.599
**55. I am not aware of the emotions I’m feeling when in conversation.**	-0.542				0.221	-0.487
**59. I can tell if I am feeling a combination of emotions at the same time.**	0.537		0.254			0.486
**13. I am often confused about the emotions that I feel.**	-0.533	0.208	0.231			-0.536
**21. I can easily label “basic emotions” (fear, anger, sadness, joy, and surprise) that I feel.**	0.480					0.528
**44. I often know what I feel but choose not to reveal it outwardly.**		0.780				0.696
**31. If I feel something, I prefer not to discuss it with others.**		0.730				0.788
**20. I often keep my emotions inside.**		0.723				0.700
**36. If I feel something, I would rather not convey it to others**		0.703				0.621
**48. If I feel something, I will convey it to others.**		-0.655			0.238	-0.689
**45. If I feel something, it often comes pouring out of me.**		-0.577				-0.466
**40. Sometimes it is good to keep my emotions to myself.**		0.484				0.389
**9. It is hard for me to talk about my complex emotions.**	-0.220	0.466				0.589
**33. I try to understand the complexity of my emotions.**			0.738			0.653
**46. I try to put effort into identifying my emotions.**			0.688			0.722
**42. I am curious about identifying my emotions.**			0.679			0.751
**34. It is important for me to acknowledge my own true feelings.**			0.665			0.692
**60. I am interested in learning about why I feel certain emotions more frequently than others.**			0.632			0.581
**12. Understanding my emotional experience is an ongoing process.**			0.544			0.340
**28. It helps me to know the reasons behind why I feel the way that I do.**			0.406			0.502
**10. When I am filled with a negative emotion, I know how to handle it.**				0.738		0.712
**6. I can still think rationally even if my emotions are complex.**				0.686		0.573
**26. I am good at controlling emotions that I do not want to feel.**				0.600		0.584
**7. I am able to wait to act on my emotions.**				0.578		0.495
**23. I am good at controlling my emotions.**	0.219	0.233	-0.217	0.538		0.740
**14. I am able to adjust my emotions to be more precise.**				0.463	0.229	0.417
**43. If a feeling makes me feel uncomfortable, I can easily get rid of it.**				0.444		0.505
**16. Knowing about my childhood experiences helps to put my present emotions within a larger context.**					0.703	0.634
**47. I can pinpoint childhood experiences that influence the way that I often think and feel.**					0.621	0.852
**1. I often think about how the emotions that I feel stem from earlier life experiences (e.g., family dynamics during childhood).**					0.587	0.660

Note. ’Principal axis factoring’ extraction method was used in combination with a ’oblimin’ rotation.

### Factor structure of the Mentalized Affectivity Scale. Confirmatory factor analysis

CFA was conducted separately on data from Sample B using the 35 items; item selection to load on CFA factors was based on EFA loadings. Table 3 presents the standardized factor loadings in Sample B. The fit of the CFA model to the data from the 248 subjects was acceptable (χ^2^ (584) = 1076.00 p < .001; RMSEA = .058; SRMR = .074). Loadings from the CFA were comparable with those found in the EFA, identifying the five factors.

### Hierarchical structure

**Fig 1:**
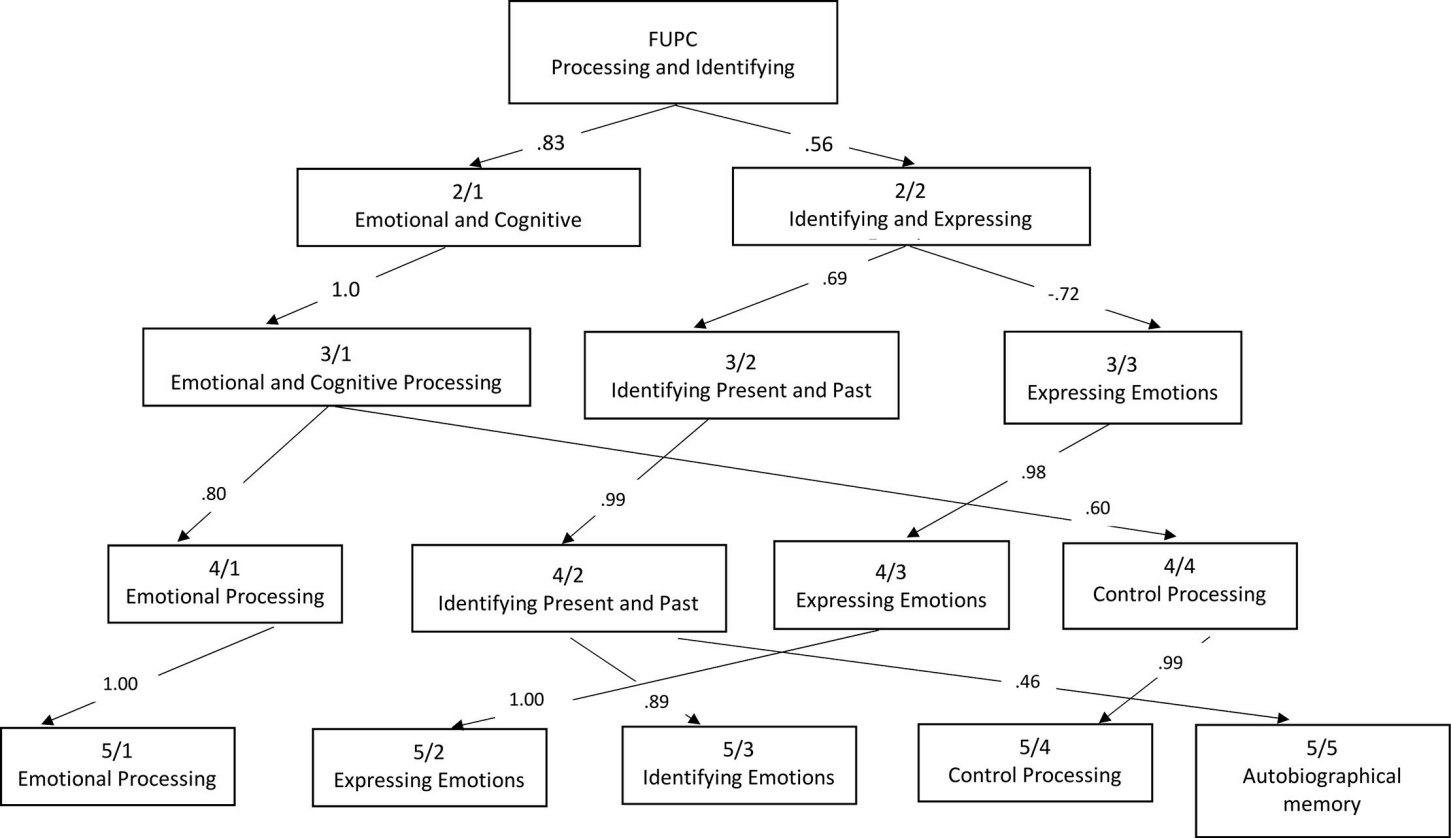
Varimax principal components derived from ratings for 35-items of the MAS. Note. The figure begins (top box) with the First Unrotated Principal Component (FUPC) and displays the genesis of the derivation of the 5 components obtained. Text within each box indicates the label of the factor. Arabic numerals within boxes indicate the number of factors extracted for a given level (numerator) and the factor number within that level (denominator; e.g., 2/1 indicates the first component in a two-component solution). Arabic numerals within the arrow paths indicate the Pearson product-moment correlation between a component obtained early in the extraction and a later component. For example, when expanding form a two-component solution to a three-factor solution (rows 2 and 3), we see that Factor 2/2, “Identifying and expressing emotions” splits into two new factors, “Identifying present and past” (which correlates .69 with the parent component) and “Expressing emotions” (which correlates -72 with the parent component).

The hierarchical structure of the one-component through five-components solution was conducted using the procedure proposed by Goldberg [39] on the total sample of participants. The resulting hierarchical structure is displayed in Fig 1. Items that loaded highest on the one-component solution (FUPC) represented Identifying and Expressing Emotions, which are related to the ability to recognize emotions and to express them, including “I am good at distinguishing between different emotions that I feel”, “I try to put effort into identifying my emotions.”, and “If I feel something, I will convey it to others”. Items in the two-components solution appeared to represent “Identifying and Processing” and “Curiosity and Expressing Emotions” dimensions of mentalized affectivity. Items that loaded high on the “Identifying and Processing” dimension were “I can quickly identify my emotions without having to think too much about it”, “I often figure out where my emotions stem from”, and “I can easily label “basic emotions” (fear, anger, sadness, joy, and surprise) that I feel”. This component remained virtually unchanged in the three-component solution. Items that loaded high on the “Curiosity and Expressing Emotions” dimension were “If I feel something, it often comes pouring out of me”, and “If I feel something, I prefer not to discuss it with others”, and "I am curious about identifying my emotions". In the three-components solution, the “Curiosity and Expressing Emotions” dimension split into two subcomponents that differentiated “Curiosity about present and past” affects from “Expressing” affects. Items that loaded highly on the “Identifying present and past” affects were "I can tell if I am feeling a combination of emotions at the same time", and "I try to put effort into identifying my emotions". Items that loaded highly on the “Expressing” dimension were “If I feel something, I prefer not to discuss it with others”, “I often know what I feel but choose not to reveal it outwardly”, “I often keep my emotions inside”. In the four-components solution, both “Curiosity about present and past” and “Expressing” dimensions remained virtually unchanged; the “Identifying and Processing” component split into two subcomponents that differentiated “Identifying” affects and “Processing” affects. Items that loaded highly on the “Identifying” were "I am good at distinguishing between different emotions that I feel", "I can quickly identify my emotions without having to think too much about it", and "I am able to understand my emotions within the context of my surroundings"; items that loaded highly on the “Processing” were "When I am filled with a negative emotion, I know how to handle it", "I am good at controlling emotions that I do not want to feel", and "I can still think rationally even if my emotions are complex". Finally, at the fifth-components solution “Identifying”, “Expressing”, and “Processing” dimensions remained unchanged. “Curiosity about present and past” split into two subcomponents that differentiated “Curiosity about emotions” and “Autobiographical memory”. Items that loaded highly on the “Identifying” were "I try to put effort into identifying my emotions", "I try to understand the complexity of my emotions", and "I am curious about identifying my emotions", and items that loaded highly on the “Autobiographical memory” dimension were "I can pinpoint childhood experiences that influence the way that I often think and feel", "Knowing about my childhood experiences helps to put my present emotions within a larger context", and "I often think about how the emotions that I feel stem from earlier life experiences (e.g. family dynamics during childhood)".

### Reliability of the Mentalized Affectivity Scale and correlations among factors

All the factor scores showed an acceptable distribution; skewness and kurtosis showed normal distribution (SkewnessMIN = -0.72-SkewnessMAX = 0.21; KurtosisMIN = -0.42-KurtosisMAX = 0.58).

The analysis of reliability performed on the data collected from all participants showed that the scale has adequate internal consistency for all factors. All Cronbach’s alphas were adequate: “Identifying Emotions” = .86, “Expressing Emotions” = .84, “Curiosity about Emotions”.82, “Processing Emotions” = .79, “Autobiographical Memory”. = .75. As long as correlations among the five factors, “Identifying Emotions” and “Curiosity about Emotions”, “Expressing Emotions” and “Processing Emotions”, “Curiosity about Emotions” and “Processing Emotions” are not linked, whereas all the other factors show significantly positive correlations.

### Convergent validity

Convergent validity was assessed with correlations among the five mentalized affectivity factors, and the reflective functioning and emotion regulation.

As it can be seen in Table 4, Identifying Emotions is positively correlated with the ERQ Cognitive reappraisal emotion regulation strategy, and with the Certainty in the reflecting functioning, and it is negatively correlated with the emotion regulation strategy of Expressive suppression and Uncertainty in reflective functioning. Expressing emotions is strongly negatively correlated with the Expressive suppression emotion regulation strategy. Curiosity about Emotions is significantly positively correlated with the Cognitive reappraisal strategy, while it is negatively correlated with the Expressive suppression strategy. Processing Emotions is significantly positively correlated with both scales of the Cognitive reappraisal strategy, and with the Certainty in the reflective functioning, while it is negatively correlated with the Uncertainty in reflective functioning. Finally, Autobiographical Memory is strongly positively correlated with the Cognitive reappraisal strategy, and it is negatively correlated with the Expressive suppression strategy.

**Table 4:** Convergent correlations with reflective functioning and emotion regulation.

	*Mentalized Affectivity Components*				
	Identifying Emotions	Expressing Emotions	Curiosity about Emotions	Processing Emotions	Autobiographical memory
**RFQ_C**	0.392***	0.052	0.037	0.379***	-0.028
**RFQ_U**	-465***	-0.075	0.030	-0.503***	0.016
**ERQ_CR**	0.129***	0.080	0.275***	0.342***	0.242***
**ERQ_ES**	-0.252***	-0.727***	-0.236***	0.126***	-0.093*

N = 506

*p < .05

***p < 0.01

Note. RFQ_C: Reflective Functioning Certainty subscale; RFQ_U: Reflective Functioning Uncertainty subscale; ERQ_CR: Emotion Regulation Cognitive reappraisal subscale; ERQ_ES: Emotion Regulation Expressive suppression subscale.

### Demographics, personality, well-being, life satisfaction and self-efficacy

Correlations among mentalized affectivity factors and the other measures are displayed in Table 5.

**Table 5:** External correlates of mentalized affectivity.

	Mentalized Affectivity Components				
	Identifying Emotions	Expressing Emotions	Curiosity about Emotions	Processing Emotions	Autobiographical memory
**Demographics**					
**Age**	0.129**	0.071	-0.058	0.196***	-0.018
**Education**	0.096*	0.143**	0.203***	0.018	0.008
**Personality**					
**Openness**	0.095*	0.181***	0.151***	0.106*	0.004
**Conscientiousness**	0.339***	0.069	0.037	0.250*	0.053
**Extraversion**	0.103*	0.386***	0.134**	0.025	0.039
**Agreeableness**	0.167***	0.022	0.188***	0.194***	0.064
**Neuroticism**	-0.289***	0.022	0.082	-0.480***	0.076
**Life Satisfaction**					
**SWLS**	0.284***	0.151***	0.031	0.253***	0.070
**Self-Efficacy**					
**GSE**	0.421***	0.104*	0.099*	0.525***	0.027

N = 506

*p < .05

***p < 0.01

#### Demographics

Identifying Emotions is moderately positively correlated with age, and weakly positively correlated with education. Expressing Emotions and Curiosity about Emotions are both significantly positively correlated with education. Finally, Processing Emotions is significantly positively correlated with age.

#### Personality

As for personality scales, Identifying Emotions is positively correlated with Conscientiousness and Agreeableness traits, it is weakly positively correlated with Openness and Extraversion traits, while it is strongly negatively correlated with Neuroticism. Expressing Emotions is strongly positively correlated with Openness and Extraversion. Curiosity about Emotions is positively correlated with Openness, Agreeableness and Extraversion traits. Processing Emotions is significantly positively correlated with Agreeableness, it is weakly positively correlated with Openness and Conscientiousness traits, and it is negatively correlated with Neuroticism. Finally, Autobiographical Memory is not correlated with the others measures.

#### Life satisfaction

Identifying Emotions, Expressing Emotions and Processing Emotions are strongly positively correlated with Life satisfaction.

#### Self-efficacy

Identifying Emotions and Processing Emotions are positively correlated with General Self-Efficacy, while Expressing Emotions and Curiosity about Emotions are weakly positively correlated with this construct.

## Discussion

The present research tested the factorial validity of the Italian version of the MAS in an Italian sample. Moreover, we tested reliability, concurrent and convergent validity by examining associations with the MAS and its socio-affective correlates, such as emotion regulation and reflective functioning. Finally, we explored possible links among mentalized affectivity as tested with the MAS and other measures of personality and well-being, such as life satisfaction and self-efficacy. Referring to the factorial structure of the Italian version of the MAS, the confirmative factor analysis did not confirm the original three-factors structure. As it can often be the case, scales translated in different languages and analyzed in different cultural contexts may not have the same latent factor structure as the original version: then, we conducted an exploratory factor analysis, followed by a new confirmative factor analysis, to examine the latent structure of the Italian version of the MAS. Following these steps, we delineated a new five-factors structure: Identifying Emotions, Expressing Emotions, Curiosity about Emotions, Processing Emotions and Autobiographical Memory.

Research has started to explore cultural differences underlying the construct of mentalized affectivity, and the growing interest in cultural differences in mentalization may provide a helpful path for the interpretation of our results. In a recent review, Aival-Naveh and colleagues [11] proposed that mentalizing development could be interpreted from different perspectives: a universalist one, that highlights the role of innate aspects of mentalization; a relativist one, that underlines the importance of the context in mentalization development; an intermediate one, that relies between the other two perspectives. This last hypothesis seems compatible with the mentalized affectivity theoretical model proposed by Greenberg and colleagues [1], because it assumes the existence of basic psychological processes, similar across cultures, which are affected during human development by specific cultural factors (a relevant cultural factor can be, for example, the possibility to establish attachment relationships in extrafamilial contexts, e.g. at school; see [43, 44].

Also following the theoretical model proposed by Greenberg and colleagues [1], the biological bases of mentalization develop during infancy and childhood through parental attachment and early social experiences, which are deeply influenced by culture, and then generate mentalized affectivity. Regarding mentalization, Aival-Naveh and colleagues [43] proposed a macro-difference between individualistic *vs*. collectivistic cultures: in individualistic/Western cultures, mentalization is mainly oriented to the self, whereas in collectivistic/Eastern cultures, mentalization abilities develop firstly with the aim to understand others. Notwithstanding the above arguments, research in this area is still limited and do not analyze specific cultures in detail. In our case, although American and Italian culture can be both considered Western cultures, i.e. individualistic cultures, it is possible to speculate about the existence of some differences in mentalistic and affective development that can have an impact on a complex skill such as mentalized affectivity. The hierarchical model that we proposed has showed that in the Italian version of the MAS structure the three original factors are already present on second level, accompanied by a fourth factor, Curiosity, that is splitted in the third level to Curiosity about Present and Past and, finally, it is divided into Curiosity about Emotions and Autobiographical memory. Both Curiosity about Emotions and Autobiographical memory factors refer to the individual’s tendency to question their present and past emotions, in particular, the role that these emotions play in their current experience; these factors are well explained by the theory of mentalized affectivity. Regarding Curiosity about emotions, Jurist [3] notes, for example, that not all people in therapy are necessarily interested in their emotions, which might predict to the duration of a therapy. Curiosity may be regarded as the basis of the mentalized affectivity; in order to become able to “being aware of one’s one affect by remaining within that affective state”, it is important to be interested in emotional experience and to consider this experience relevant to oneself. At the same time, curiosity may relate to the present or the past, so the individual may develop an interest in understanding how her/his previous experiences impact her/his current emotional state. According to Greenberg and colleagues [1], autobiographical memory can serve to illuminate how past emotional experience has an impact upon current experience, a critical element of mentalized affectivity. We may speculate that the cultural differences between Italy and the US influence the styles of how people reflect on the past and consider such reflections useful for understanding the present. In fact, European cultures and education stress the value of knowledge of the past: children and adolescents read classical texts, in their mother language or in the original language (i.e., in Latin), that often focus on the relationship between the inner world of the characters and their behavior (think of the Homeric classics, or the Romantics). Moreover, history is really important because educators believe that it is only possible to understand the present through knowledge of one’s own origins. We can suppose that US culture and education are different, more oriented to the present and to the future: wide space is given to the study of technology and the continuous impulse to innovate are promoted as part of this culture and education, which tends to deemphasize the need for revisiting the past. For these reasons, it is possible that Italians are more used to questioning their emotions and interpreting the present in light of the past than Americans [45]. So, the Italian version of the MAS seems to represent a detailed description of the mentalized affectivity dimensions, as it suggests two new factors that had so far only been hypothesized in the literature.

In order to assess the reliability and the concurrent and convergent validity, we examined the links between the MAS factors and emotion regulation and reflective functioning constructs, confirming our hypothesis. In fact, referring to emotion regulation, results showed positive correlations between the cognitive reappraisal and four of the five MAS factors, Identifying Emotions, Curiosity about Emotions, Processing Emotions and Autobiographical Memory: on the contrary, the link between cognitive reappraisal and expression of emotions is not present. We can assume that all the factors correlated with cognitive reappraisal refer to internal abilities of the individual, that is, being able to identify, to be curious, to process (as suggested by Greenberg and colleagues [1] and to use autobiographical memory, without expressing those mental states externally. It is possible that the Expressing Emotions component of the mentalized affectivity comes into play when the emotion must not only be thought, as in the case of the cognitive reappraisal, but also communicated to others, i.e. in the interpersonal sphere.

As regards the construct of reflective functioning, our results provide evidence that both emotional and cognitive dimension of processing evaluated in the MAS are positively related to the tendency to hyper-mentalize and negatively related to the tendency to hypo-mentalize. In the theoretical perspective proposed by Greenberg and colleagues [1], the processing dimension indicates the tendency to modulate, refine and regulate emotions, i.e. the tendency to think about emotions, a trait directly involved in ability to mentalize well. We can assume that people with an “over-mentalizing” attitude are able to focus on emotions, in terms of awareness and control, i.e. Processing, whereas people with a “hypo- mentalizing” attitude are not focused on their own emotions, so that they are not able to recognize and manage these internal states.

Moreover, we found several links among four out of the five MAS factors and the others constructs examined in this study. As regards personality, the results showed that Identifying Emotions and Processing Emotions are positively related to almost all the personality characteristics, and negatively with Neuroticism. Moreover, the Expressing and Curiosity components of mentalized affectivity are related to the Openness, Extraversion and Agreeableness personality characteristics. Although the link between mentalization and personality is already well-established in the literature, especially pertaining to personality disorders (just think about borderline personality disorder; [6, 46], recently Karterud and Kongerslev [47] proposed the Temperament-Attachment-Mentalization-Based theory of personality: the above-cited constructs represent innate or experiential components of the personality, intrinsically linked to each other, as they contribute to personality construction through emotion regulation abilities. So, this theory aims at explaining both typical and pathological personality in a structure similar to that of mentalized affectivity: in both cases, the authors assume the existence of inner developmental bases that allow the subject to live relational experiences, such as attachment relationships, that impact on their her/his ability to manage emotions. We can also hypothesize that mentalized affectivity is a fundamental aspect of adult personality, which derives precisely from the encounter of mentalizing and emotion regulation skills. At the same time, correlations among four out of the five factors of the MAS (excluding Autobiographical Memory), life satisfaction and self-efficacy confirm, as in Greenberg and colleagues [1], the important role of mentalized affectivity in individual well-being: being curious about emotions and being able to properly process, identify and express emotions allow the understanding of oneself and of others, favoring individual well-being and the ability to face life effectively.

The lack of links between the Autobiographical Memory factor and the others scales is not surprising: although personal memories play an important role in mentalized affectivity, we have to remember that this construct is mainly oriented to the present, because it refers to the capacity of being aware of one’s one affect by remaining within that affective state [6]: probably, when responding to questions about personality, life satisfaction and self-efficacy, people tend to refer to their present experience and to put their past ones on a back burner.

As far as personal information is concerned, Identifying Emotions and Processing Emotions factors correlate with age. As the literature argues [1–3], mentalized affectivity is an adult ability, and we can suppose that Identifying and Processing Emotions are two complex factors of this construct, improving with age. Items composing the Identifying Emotions factor seek to recognize the link between the emotional experience and the context, or to individuate the origin of the emotion, both operations involving highly cognitive activity. At the same time, Processing Emotions integrates emotional and cognitive skills, requiring a high level of self-awareness. Therefore, it may be conceivable that precisely these components of mentalized affectivity are the most apparent in adulthood.

With regards to the structural characteristics of our sample, we found a correlation between educational level and Identifying Emotions, Expressing Emotions and Curiosity about Emotions factors. These three factors involve the ability and the propensity to think about one’ s own emotions and to reflect on their origin, to monitor oneself and one’s own abilities, and to name emotions appropriately: all these activities are related to the metacognitive and self-regulatory reasoning, as well as to linguistic skills. People with a high level of education develop more advanced metacognitive and self-regulatory skills, and are more articulate than those who have less years of study; this can affect their propensity to be aware of their emotions, how to name and manage them properly. At the same time, people with lower self-regulation skills may be less likely to reach a high level of education, so in adulthood they may struggle to apply metacognitive strategies to reason about emotions. Finally, usually age and educational level are associated, and this can explain the double correlation between Identifying Emotions, age and education: the older a person is, the more elevated their level of education is, prompting them to have more cognitive tools to engage in identifying their emotions. Among the mental features related to the age that facilitate the identification of emotions we can mention the "cognitive reserve” [48], which emphasizes the role of individual differences in cognitive coping with emotional and mental burdens, helping people to become increasingly resilient to external stressors.

This paper has also several limits that need to be considered. First, compared with the original sample, the Italian sample is smaller and does not involve clinical information of the participants. In the future, it will be interesting to test the validity of the MAS also in a clinical sample, in order to compare the Italian data with the original ones (for the relevance of the mentalization in clinical samples see, for example [49, 50]. Moreover, in this research the educational level of the sample is different from the original one: to pursue our speculation about cultural differences as a way to interpret the new factors that emerged, it would be desirable to compare a sample similar on this variable. In addition, in accordance with the original research, our online survey did not inquire about the specific region where the participants live: although we aimed to compare the US and Italian populations, it is possible that knowing the specific area of life of Italian participants belong could provide additional relevant information. With respect to the measures used, we wanted to remain aligned to the original paper, so we evaluated personality characteristics with the Italian validation of the TIPI. This test shows low reliability values, similar to that of the original version [27]: the reliability seems to be a limit of the scale structure, it is possible that the use another measure of personality might have provided more accurate and consistent results with respect to this construct.

## Conclusion

In this paper we proposed the validation of the Italian version of MAS, and we found a more articulated factorial structure than the original scale. Specifically, the new factors of Curiosity about Emotions and Autobiographical Memory emerged, thus highlighting to important components of mentalized affectivity that in our sample is well distinguished from the other ones. We also verified the validity of this factorial structure, and we confirmed the relationship of the mentalized affectivity construct with other psychological correlates, highlighting the role of mentalized affectivity in individual well-being. Taken altogether, our findings show that the Italian version of the MAS could be considered a useful tool in the Italian context, both for research activities and clinical practices, enriching the complexity of the construct of mentalization and the variety of tasks devised to test such a critical ability for social life.

## Supporting information

S1 Data (SAV)
