# Correction: The Mentalized Affectivity Scale (MAS): Development and validation of the Italian version

**DOI:** 10.1371/journal.pone.0257989

**Published:** 2021-09-23

**Authors:** Teresa Rinaldi, Ilaria Castelli, Andrea Greco, David M. Greenberg, Elliot Jurist, Annalisa Valle, Antonella Marchetti

There are errors in the abstract. The line “The hierarchically structured MAS factors are: Emotional Processing (being able to process emotion in situations); Expressing Emotions (talking and knowing emotions); Identifying Emotions (awareness of emotions); Control Processing (to control emotional reactions and expression), and Autobiographical Memory (related to childhood experiences).” should instead read as follows: “The hierarchically structured MAS factors are: Identifying Emotions (awareness of emotions); Expressing Emotions (talking and knowing emotions); Curiosity about Emotions (Reflecting on own emotional experience to find a meaning); Processing Emotions (to control emotional reactions and expression), and Autobiographical Memory (related to childhood experiences).”

Additionally, Fig 1 is incorrect. The authors have provided a corrected version here.

**Fig 1:**
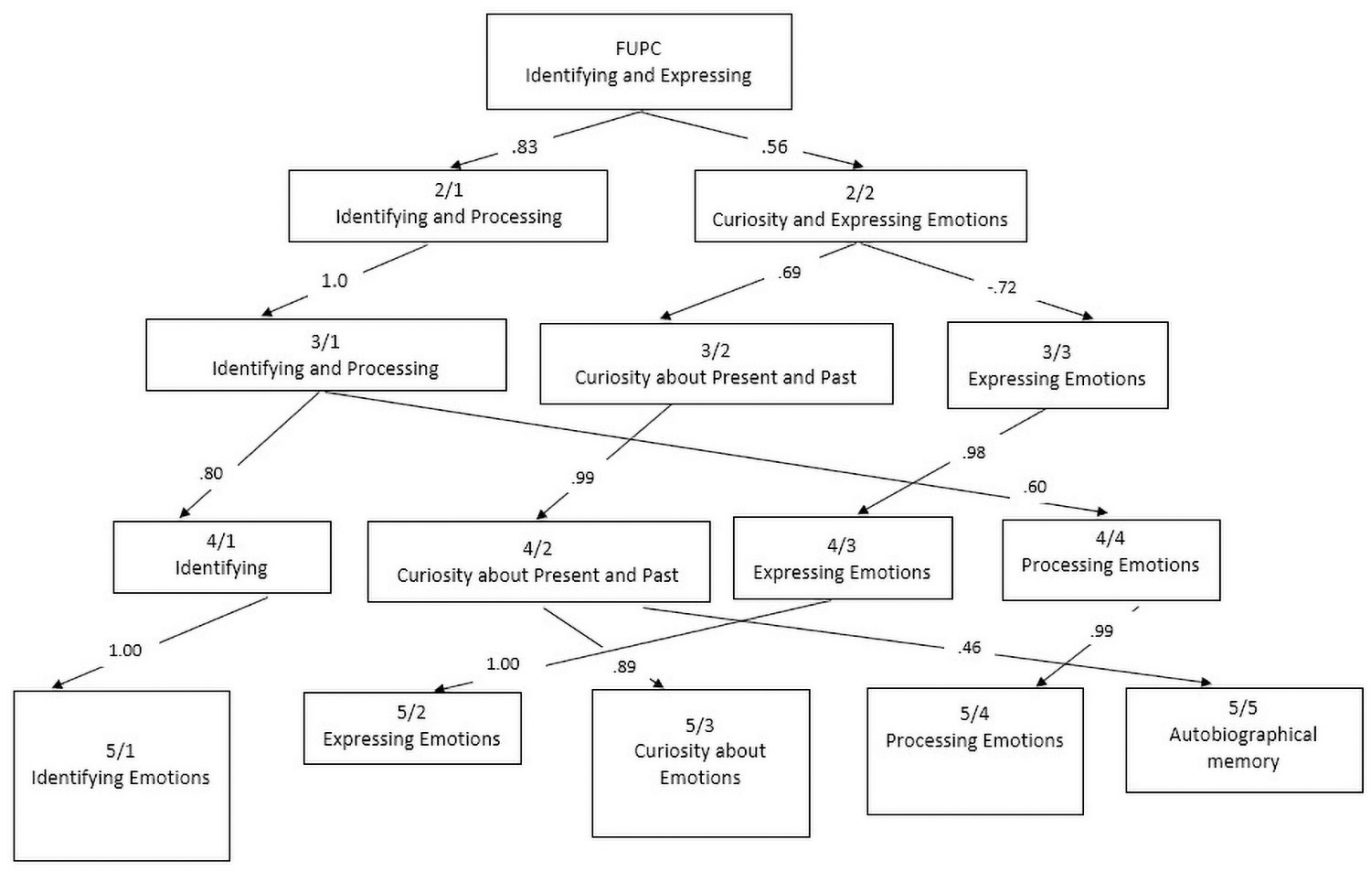
Varimax principal components derived from ratings for 35-items of the MAS. Note. The figure begins (top box) with the First Unrotated Principal Component (FUPC) and displays the genesis of the derivation of the 5 components obtained. Text within each box indicates the label of the factor. Arabic numerals within boxes indicate the number of factors extracted for a given level (numerator) and the factor number within that level (denominator; e.g., 2/1 indicates the first component in a two-component solution). Arabic numerals within the arrow paths indicate the Pearson product-moment correlation between a component obtained early in the extraction and a later component. For example, when expanding from a two-component solution to a three-factor solution (rows 2 and 3), we see that Factor 2/2, “Curiosity and Expressing Emotions” splits into two new factors, “Curiosity about Present and Past” (which correlates .69 with the parent component) and “Expressing emotions” (which correlates -72 with the parent component).
